# Prospective validation protocol for artificial intelligence-assisted standardization of Coma Recovery Scale-Revised scores using electroencephalography and clinical data in disorders of consciousness

**DOI:** 10.3389/fmed.2026.1904678

**Published:** 2026-08-03

**Authors:** Chuangan Zhou, Yuzhu Gao, Jinqiu Shan, Yida Dai, Yanfei Zhuang, Dazhong Sun, Xiangfeng Chi

**Affiliations:** 1 Guangdong Provincial Second Hospital of Traditional Chinese Medicine, Guangzhou, Guangdong, China; 2 Guangzhou University of Chinese Medicine, Guangzhou, Guangdong, China; 3 The Fifth Clinical College of Guangzhou University of Chinese Medicine, Guangzhou, Guangdong, China

**Keywords:** artificial intelligence, clinical validation, Coma Recovery Scale-Revised, disorders of consciousness, electroencephalography, SPIRIT-AI, study protocol

## Abstract

**Background:**

Disorders of consciousness (DoC) after severe acquired brain injury are difficult to diagnose at the bedside. The Coma Recovery Scale-Revised (CRS-R) is the behavioral reference standard, but raw scores can be affected by arousal fluctuation, motor impairment, sensory deficits, sedation, language disturbance, and local assessment conditions.

**Objective:**

This protocol describes a prospective validation study of a multimodal artificial intelligence (AI) system designed to standardize CRS-R scores by integrating routine electroencephalography (EEG), bedside CRS-R subscale scores, and selected clinical contextual variables against an adjudicated standardized reference.

**Methods and analysis:**

The study includes retrospective model development followed by prospective, blinded clinical validation at Guangdong Second Traditional Chinese Medicine Hospital. The locked model will use subscale-specific ordinal prediction modules and a rule-constrained integration layer to generate AI-standardized CRS-R subscale scores, total scores, diagnostic categories, uncertainty estimates, and prespecified case-level explanations. Prospective participants will undergo two independent bedside CRS-R assessments and routine scalp EEG within target and maximum assessment windows. A blinded expert committee will generate the reference CRS-R score using paired behavioral records, available videos, nursing observations, medication and confounder records. The primary endpoint is the participant-level reduction in absolute CRS-R total-score error after AI-assisted standardization relative to the adjudicated reference. Secondary endpoints include subscale agreement, diagnostic-category agreement, inter-rater variability, uncertainty-flag performance, workflow feasibility, and exploratory day-14 consciousness improvement.

**Ethics and dissemination:**

The protocol was approved by the Ethics Committee of Guangdong Second Traditional Chinese Medicine Hospital (approval number Z202603-004-01; review date March 25, 2026; approval validity March 27, 2026 to March 26, 2027). Results will be disseminated through peer-reviewed publication and academic presentations.

**Clinical Trial Registration:**

https://www.chictr.org.cn, Chinese Clinical Trial Registry, ChiCTR2600124984.

## 1 Introduction

Disorders of consciousness (DoC) after severe acquired brain injury are diagnostically challenging and ethically consequential. Judgments about residual consciousness influence prognosis, rehabilitation referral, treatment escalation, and goals-of-care discussions (1–3). Current frameworks still place standardized behavioral examination at the center of DoC assessment, while acknowledging that electrophysiological and neuroimaging biomarkers have not yet been fully translated into routine bedside care (1, 3–5).

This gap matters clinically. In a prospective *New England Journal of Medicine* study, 60 of 241 patients without observable command-following (25%) showed cognitive motor dissociation on task-based EEG or functional magnetic resonance imaging (6). A critical care review estimated covert consciousness in approximately 15%–20% of ICU patients who appear behaviorally unresponsive (7). Sleep-spindle and multimodal biomarker studies further show that electrophysiological signals may precede or complement behavioral signs of recovery (8–10).

The CRS-R remains the behavioral reference standard, but raw scores are vulnerable to factors that can obscure purposeful behavior. These include aphasia, severe motor impairment, sensory limitation, fluctuating arousal, sedative exposure, body position, and inter-rater variation (1, 3, 11, 12). Repeated assessments are often needed because first-assessment misclassification remains common, and CRS-R thresholds may not perform identically across subscales, etiologies, and clinical settings (13–18).

EEG is attractive for CRS-R score standardization because it is bedside-compatible, repeatable, and increasingly supported by mature analytic pipelines. Neuromonitoring reviews also emphasize that continuous EEG and related bedside modalities can help detect neurological events that are otherwise difficult to observe in sedated or intubated patients (19). Recent studies have used deep learning, interpretable temporal-spectral modeling, dynamic EEG pattern analysis, and low-dimensional EEG representations to detect awareness or predict outcome in DoC (5, 20–26). Multimodal studies also suggest that behavior, electrophysiology, imaging, and clinical context provide complementary rather than interchangeable information (4, 27–29).

Most AI studies in DoC still focus on retrospective state classification or outcome prediction. A 2025 scoping review identified only 21 eligible peer-reviewed AI studies after screening 49,417 records, and recent reviews note that many models classify unresponsive wakefulness syndrome (UWS) vs. minimally conscious state (MCS) instead of preserving CRS-R subscale structure or prospectively validating human-in-the-loop deployment (11, 20, 30, 31). Prospective DoC research also requires explicit case ascertainment, outcome definitions, covariate capture, and timing of data acquisition because behavioral responsiveness is subtle and fluctuating (32). This protocol addresses that gap by evaluating whether an EEG-clinical AI system can standardize continuous CRS-R total and subscale scores against an adjudicated standardized reference.

In this protocol, AI-assisted CRS-R score standardization means generation of an adjusted subscale profile and total score using a locked model that incorporates raw bedside CRS-R subscale scores, EEG-derived features, and predefined contextual variables. The term does not refer to statistical calibration of predicted probabilities against observed outcome frequencies. The system is intended to support standardized review of bedside scores during validation, not to replace CRS-R examination or expert clinical judgment.

The study is reported as a prospective Study Protocol and is aligned with SPIRIT and SPIRIT-AI principles for protocols involving artificial intelligence (33). A completed SPIRIT and SPIRIT-AI checklist is provided as Supplementary Table S1. The protocol is also informed by TRIPOD+AI reporting principles for prediction model development and evaluation (34). It is designed to test a locked model under blinded, real-world clinical validation conditions rather than to replace clinician judgment during the validation stage.

## 2 Methods and analysis

### 2.1 Study design and setting

This is a two-stage study consisting of retrospective model development followed by prospective, blinded clinical validation. The study will be conducted at Guangdong Second Traditional Chinese Medicine Hospital. The clinical research unit named in the ethics approval is Guangdong Second Traditional Chinese Medicine Hospital, with implementation through the Department of Acupuncture and Rehabilitation, Huangpu Hospital District.

The primary aim is to determine whether AI-standardized CRS-R total scores show smaller absolute error than unstandardized bedside CRS-R total scores when compared with an adjudicated standardized reference. The secondary aims are to assess subscale-level agreement, diagnostic-category agreement, inter-rater variability, uncertainty-flag performance, workflow feasibility, and exploratory day-14 consciousness improvement.

### 2.2 Study population

**Figure 1:**
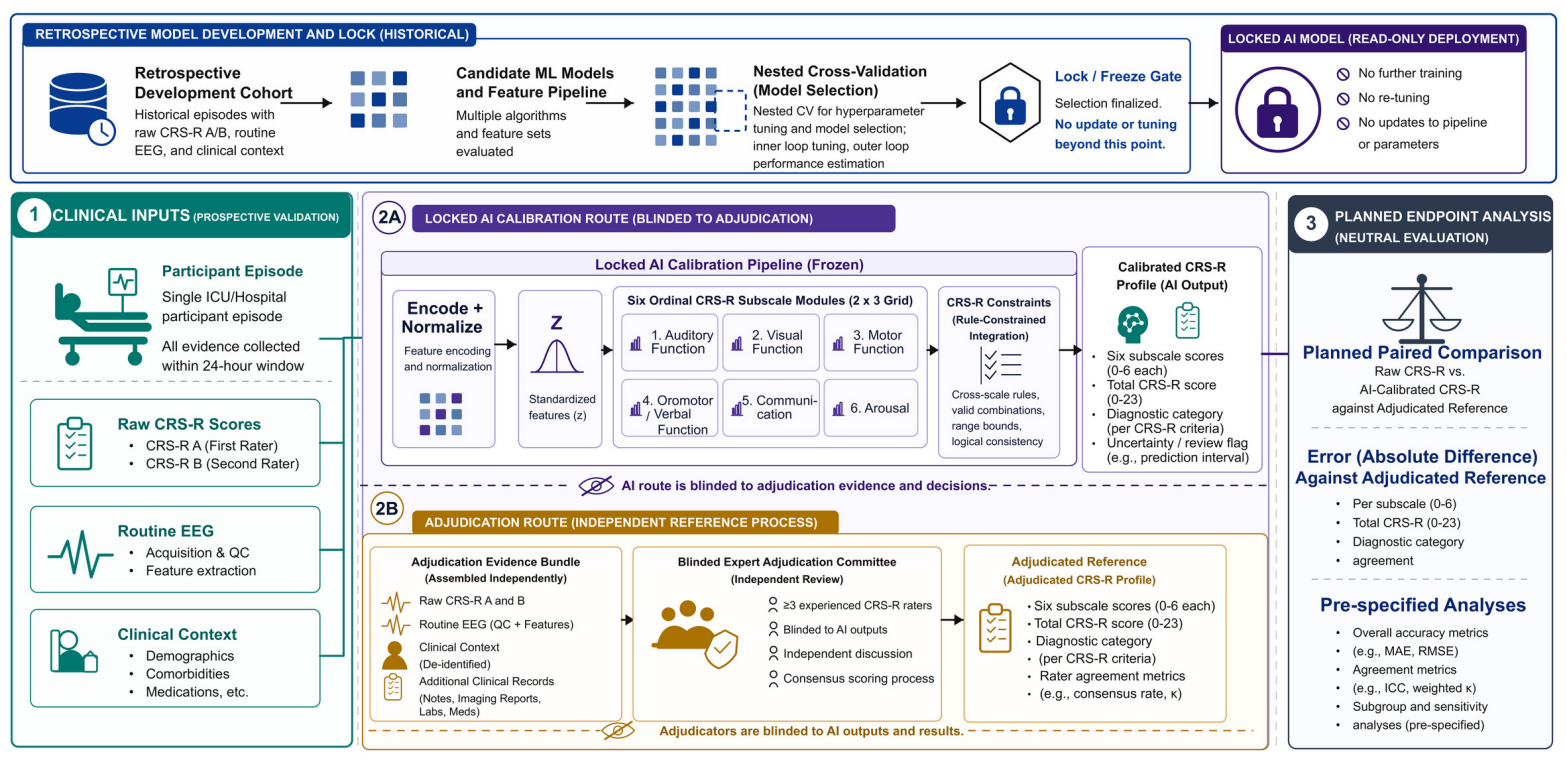
Study design and route-separated validation framework for AI-assisted CRS-R score standardization.

Adult inpatients with acquired disorders of consciousness will be screened during the recruitment period. Whenever applicable, the retrospective model-development and prospective-validation cohorts will follow the same prespecified inclusion and exclusion criteria, ensuring clinical alignment across study phases. Figure 1 summarizes the overall pathway from participant screening and multimodal data collection to locked AI-assisted score standardization, blinded adjudication, and endpoint analysis.

#### 2.2.1 Inclusion criteria

Participants must meet all of the following criteria:

1. Age 18–80 years.
2. Severe acquired brain injury based on clinical history, imaging, and presentation, including traumatic brain injury, hypoxic-ischemic encephalopathy after cardiopulmonary resuscitation, extensive intracerebral hemorrhage, or ischemic stroke.
3. Clinical stability sufficient for bedside CRS-R assessment and routine scalp EEG recording.
4. Behavioral diagnosis within the DoC continuum, including coma, UWS/vegetative state, MCS minus, or MCS plus, according to accepted neurological guidelines.
5. Ability to complete two independent CRS-R assessments and one EEG recording within the protocol assessment window.
6. Written informed consent provided by the legally authorized representative or immediate family member.
7. Stable vital signs, absence of severe cardiopulmonary failure, and the ability to safely suspend continuous intravenous sedation within the assessment window when clinically permissible.

#### 2.2.2 Exclusion criteria

Participants will be excluded if any of the following applies:

1. Persistent deep sedation, general anesthesia, or uncontrolled status epilepticus during the assessment window.
2. Complete paralysis from severe cervical spinal cord injury or peripheral neuromuscular blockade that prevents interpretation of CRS-R motor or oral motor subscales.
3. Severe irreversible neurodegenerative disease or severe psychiatric disorder that would confound DoC diagnosis.
4. EEG dominated by uncontrolled epileptiform activity or status epilepticus, preventing valid resting-state background or connectivity feature extraction.
5. EEG recordings of insufficient quality despite repeat acquisition when repeat acquisition is clinically feasible.
6. Massive bilateral skull defects preventing accurate and symmetrical scalp electrode placement according to the international 10–20 system.
7. Concurrent severe systemic infection or metabolic disorder causing an acute, reversible decline in consciousness.
8. Refusal or withdrawal of informed consent by the legally authorized representative.

### 2.3 Retrospective model development

The retrospective phase will establish the preprocessing pipeline, feature definitions, model architecture, missing-data rules, quality-control thresholds, and interpretability outputs before prospective enrollment begins. The model will be fully trained and locked before the first prospective participant is included.

#### 2.3.1 Clinical contextual variables

Clinical contextual variables will be extracted from standardized electronic case report forms. These variables will include age, sex, etiology, time since injury, body position during assessment, tracheostomy or endotracheal tube status, mechanical ventilation, recent sedative exposure, antiseizure medication exposure, fever or active infection, metabolic derangement, recent seizures, severe motor impairment, aphasia or suspected language impairment, major visual or auditory limitation, and the time interval from recent acupuncture or neurorehabilitation intervention. The case report form will record the timing of CRS-R assessment, EEG acquisition, treatment sessions, nursing procedures, airway events, and clinically important changes in arousal state. These variables are included to account for known sources of behavioral score variability and to support reproducible covariate capture in prospective DoC research (32).

#### 2.3.2 Sedation and medication handling

Sedation exposure will be documented before each CRS-R assessment and EEG recording. Continuous intravenous sedatives will include propofol, midazolam or other benzodiazepines, dexmedetomidine, opioid infusions, and other locally used sedative or analgesic agents. Intermittent sedatives, rescue analgesics, neuromuscular blocking agents, and antiseizure medications will be recorded separately. For each agent, the electronic case report form will capture current administration status, stop time when applicable, dose in the preceding 24 h, and administration within 6 h before the assessment episode.

CRS-R assessment and EEG acquisition will be attempted only when the treating team judges that temporary reduction or suspension of continuous sedation is clinically permissible. The target condition is absence of continuous intravenous sedative infusion during the behavioral assessment and EEG recording. Minimum target washout intervals will be 30 min after stopping propofol or dexmedetomidine infusions, 30 min after short-acting opioid boluses or infusion reduction, and 4 h after benzodiazepine bolus administration or midazolam infusion discontinuation when clinically permissible. Longer intervals will be recorded when hepatic or renal dysfunction, prolonged infusion, or delayed awakening is present. Antiseizure medications will not be withheld for study purposes; recent loading doses, rescue therapy, or major dose changes within 24 h will be recorded. Sedation depth will be recorded using the Richmond Agitation-Sedation Scale, FOUR score arousal observations, or the closest locally available structured arousal assessment. Cases with persistent deep sedation, general anesthesia, neuromuscular blockade that prevents behavioral interpretation, or major medication changes between paired ratings will be retained in feasibility summaries but excluded from prespecified sensitivity analyses when the change is judged to compromise CRS-R interpretability.

#### 2.3.3 EEG acquisition and preprocessing

Routine scalp EEG will be recorded using the international 10–20 system with at least 19 channels. The minimum sampling rate will be 256 Hz, with higher sampling rates preserved when available. The target recording duration will be at least 20 min. The target analysis segment will be an eyes-open or resting unstructured bedside recording obtained without active command-following tasks. If eyes-open recording is not feasible, the observed eye state, arousal condition, recent stimulation, and nursing or respiratory events will be documented.

EEG preprocessing will use a prespecified locked pipeline: channel inspection, removal of non-physiological or disconnected channels, bad-channel interpolation when appropriate, band-pass filtering from 0.5 to 45 Hz, 50-Hz notch filtering when line noise is present, common average or linked-mastoid re-referencing according to the locked acquisition montage, artifact detection and rejection, and extraction of artifact-reduced analysis epochs. Quantitative analysis will use 4-s epochs with 50% overlap. The minimum analyzable duration will be 5 min of artifact-reduced EEG. Epochs will be rejected when any channel shows absolute amplitude greater than 500 μV, flat signal below 1 μV for at least 5 s, abrupt step change greater than 200 μV, visually confirmed electrode-pop or movement artifact, or high-frequency muscle contamination defined as 20–45 Hz power more than three standard deviations above the recording median. Recordings with more than 30% bad channels or less than 5 min of acceptable EEG after artifact rejection will be classified as quantitative-EEG failures. The final software implementation, reference montage, and quality-control log format will be archived with the locked preprocessing package before prospective enrollment.

Quantitative features will include regional and global relative band power, alpha-delta ratio, spectral entropy, Hjorth parameters, and connectivity metrics such as coherence or phase-based synchronization indices, depending on data quality. Exploratory feature engineering during retrospective development may include variational mode decomposition, sample entropy, spectral entropy, skewness, kurtosis, and weighted phase-lag index features (35, 36). Sleep-like EEG, low-arousal recordings, stimulation exposure, and EEG quality metrics will be retained as contextual or quality variables for feasibility and sensitivity analyses rather than silently excluded.

#### 2.3.4 Model architecture

The standardization system will use a two-stage multimodal architecture. In the first stage, six subscale-specific ordinal prediction modules will estimate probability distributions over the permissible score levels for each CRS-R subscale. In the second stage, a rule-constrained integration layer will assemble the AI-standardized subscale profile, compute the standardized total score, derive the diagnostic category, and estimate prediction uncertainty using locked ensemble dispersion or conformal uncertainty logic.

This structure replaces a simple total-score regression model because it better preserves CRS-R subscale structure and diagnostic rules. Extreme Gradient Boosting, random forest, support vector regression or classification variants, and related ensemble models may be evaluated during retrospective development. The final deployed model will be selected and locked before prospective validation.

#### 2.3.5 Internal validation and model locking

During retrospective development, model performance will be assessed using nested fivefold cross-validation with patient-level splitting. If a patient contributes more than one assessment episode, all episodes from that patient will remain in the same fold. The inner loop will tune hyperparameters on the training partition, and the outer loop will estimate generalization performance on held-out patients. EEG preprocessing decisions that use data-derived thresholds, feature scaling, imputation, feature selection, dimensionality reduction, model selection, uncertainty thresholds, and score-shift review thresholds will be learned or fixed within the training partitions only and then applied to the corresponding held-out fold. Model parameters, preprocessing rules, feature definitions, missing-data handling logic, uncertainty thresholds, review thresholds, software versions, and deployment checksums will be archived before prospective use. No parameter updating, threshold tuning, or online learning will be allowed during prospective validation.

### 2.4 Prospective clinical validation

The prospective phase will evaluate the agreement between AI-standardized scores and blinded expert reference scores in a real-world neurological rehabilitation workflow. During validation, the system will operate in read-only mode. It will not replace bedside CRS-R assessment and will not determine treatment allocation.

#### 2.4.1 Rater training, standardization, and blinding

Bedside raters will be rehabilitation physicians or therapists who have completed structured CRS-R training before study initiation. Before formal enrollment, raters will complete a study-specific rater standardization phase including manual review, practice scoring, and pilot paired assessments in non-study cases. Raters must demonstrate substantial agreement during the pilot phase before participation.

For each participant, two bedside raters will be blinded to each other's scores and to AI output. EEG readers will be blinded to CRS-R scores and AI output. The adjudication committee will be blinded to rater identity, raw AI output, and model explanation results. The statistical team will analyze a de-identified dataset after database lock.

#### 2.4.2 Assessment workflow

Each participant will undergo a prespecified assessment episode. The target workflow is completion of both CRS-R assessments and the EEG recording during the same morning block and before acupuncture, neuromodulation, or intensive rehabilitation sessions. The target interval between the two CRS-R assessments is no more than 2 h, and the target EEG timing is within 2 h before or after the midpoint of the paired CRS-R assessments. If target timing is not feasible, the maximum permitted interval will be 4 h between the two CRS-R assessments and 6 h between EEG and the paired CRS-R assessment window. Reasons for exceeding the target interval will be recorded. Sedation changes, fatigue, nursing care, airway events, acute complications, and rehabilitation sessions occurring within the assessment window will be recorded and used in sensitivity analyses.

The workflow will include:

1. Eligibility confirmation and consent from the legally authorized representative.
2. Collection of baseline demographic and clinical contextual variables.
3. First bedside CRS-R assessment by rater A.
4. Second independent bedside CRS-R assessment by rater B, with a target interval of no more than 2 h and a maximum interval of 4 h after the first assessment.
5. Routine scalp EEG, with a target timing within 2 h of the midpoint of the paired CRS-R assessments and a maximum timing within 6 h of the paired assessment window.
6. Video recording of bedside behavioral assessments when consent and local policy permit.
7. Offline AI inference using each rater's raw CRS-R subscale scores, EEG data, and clinical contextual data.
8. Blinded expert adjudication to generate the standardized reference CRS-R score and diagnosis.

The order of raters will be alternated when operationally possible. Major medication, sedation, body-position, ventilatory, arousal-state, fatigue, nursing-care, treatment-session, or acute-complication changes between ratings will be documented. Participants with major instability between raters will remain in feasibility summaries and may be excluded from prespecified sensitivity analyses.

### 2.5 Reference standard adjudication

The reference standard will be an adjudicated standardized CRS-R score generated by a three-member expert committee composed of senior clinicians experienced in DoC assessment. The committee will review a de-identified package containing synchronized video recordings when available, structured bedside observation forms, nursing observations within the surrounding 12-h window, medication and airway records, body-position records, confounder documentation, and other behaviorally relevant clinical notes.

Each expert will independently rescore the CRS-R at item and subscale level according to standard CRS-R administration and scoring rules. The adjudicated reference score will not be an expert modification of CRS-R scoring. Instead, the committee will identify the highest CRS-R-consistent response observed across paired assessments and supporting records after excluding responses judged artifactual, reflexive, or confounded by temporary clinical factors.

For this protocol, a response will be considered reproducible when it is observed more than once within the paired assessment record, is corroborated by video or structured bedside documentation, or is confirmed by consensus review as a stable response pattern rather than an isolated artifact. A response will be considered behaviorally credible when it is temporally linked to the relevant CRS-R stimulus or command, is compatible with the patient's motor and sensory capacities, and cannot be better explained by reflex activity, startle, seizure activity, airway manipulation, sedation fluctuation, or another documented confounder. If independent expert scores differ by more than 2 total points or produce different diagnostic categories, a consensus meeting will be held. If disagreement remains unresolved and the participant is clinically stable, an additional senior bedside assessment within 24 h may be used as a tie-breaker.

### 2.6 Inference workflow

The complete step-by-step prospective inference pipeline of the AI standardization system is outlined in Algorithm 1. Case-level explanations will be generated with a prespecified additive feature-attribution method after prediction. These explanations will support error review and model transparency but will not be described as part of the model architecture itself.

Algorithm 1 Prospective inference workflow for AI-assisted CRS-R score standardization. 
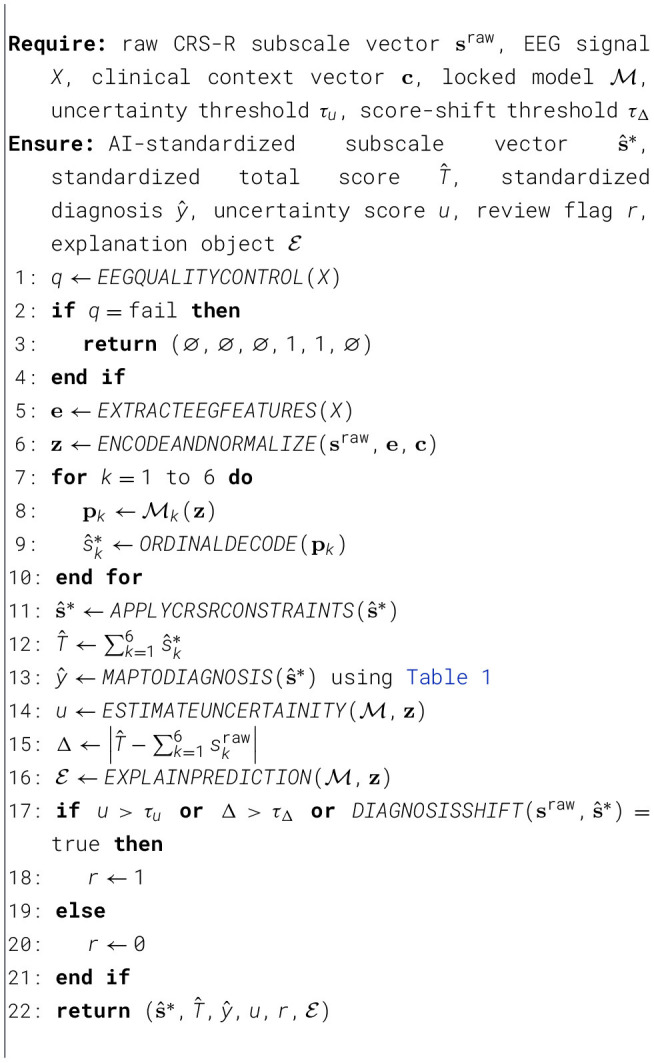

**Table 1:** Operational CRS-R diagnostic mapping used by the rule-constrained integration layer.

Diagnostic category	Operational mapping rule
Coma	No eye opening and no reproducible CRS-R evidence of wakefulness or awareness, after excluding confounding sedation, paralysis, or acute medical instability
UWS/vegetative state	Wakefulness is present, typically with eye opening or sleep-wake cycling, but CRS-R items show no reproducible evidence of purposeful behavior meeting MCS or emergence criteria
MCS minus	Reproducible low-level but purposeful CRS-R behaviors are present, such as visual pursuit, fixation, localization to noxious stimulation, automatic motor response, or object manipulation that does not meet MCS plus criteria
MCS plus	Reproducible higher-order CRS-R behaviors are present, including command following, intelligible verbalization, or intentional communication that is not yet functional communication
Emergence from MCS	Functional communication or functional object use is present according to standard CRS-R rules

### 2.7 Outcome measures

The primary outcome is the participant-level reduction in absolute CRS-R total-score error after AI-assisted standardization. For rater *j* of participant *i*, raw and AI-standardized absolute error, and participant-level paired difference are defined in Equations 1, 2, and3, respectively.


$$\begin{array}{l} \Delta_{ij}^{\text{raw}}=|T_{ij}^{\text{raw}}-T_{i}^{\text{ref}}|, \end{array}$$ (1)

$$\begin{array}{l} \Delta_{ij}^{\text{AI}}=|{\hat{T}}_{ij}-T_{i}^{\text{ref}}|, \end{array}$$ (2)

where $T_{i}^{\text{ref}}$ is the adjudicated reference total score.

The participant-level paired difference will be:


$$\begin{array}{l} D_{i}=\frac{1}{2}\overset{2}{\underset{j=1}{\sum }}\left(\Delta_{ij}^{\text{raw}}-\Delta_{ij}^{\text{AI}}\right). \end{array}$$ (3)

The primary analysis will test whether the mean of *D*_*i*_ differs from zero, with positive values indicating error reduction after AI-assisted standardization. This definition aligns the endpoint with the participant-level recruitment target while preserving both rater-specific observations.

Secondary outcomes include mean absolute error, root mean square error, agreement between AI-standardized and reference diagnostic category, agreement between standardized and reference subscale scores, reduction in inter-rater variability, sensitivity and specificity for detection of MCS and emergence from MCS, macro-averaged F1-score, uncertainty-flag performance, workflow feasibility, and exploratory consciousness improvement at day 14. Consciousness improvement will be defined as at least one ordered diagnostic-category improvement by day 14 compared with baseline. CRS-R total-score change at day 14 will be analyzed as a sensitivity metric. Discharge status and discharge timing will be reported descriptively rather than used as a primary prognostic time point.

Inter-rater variability for raw and AI-standardized total scores is calculated as specified in Equations 4 and 5.


$$\begin{array}{l} V_{i}^{\text{raw}}=|T_{i1}^{\text{raw}}-T_{i2}^{\text{raw}}|, \end{array}$$ (4)

$$\begin{array}{l} V_{i}^{\text{AI}}=|{\hat{T}}_{i1}-{\hat{T}}_{i2}|. \end{array}$$ (5)

### 2.8 Error analysis

Prediction errors will undergo prespecified review. A serious prediction discrepancy event will be defined as an absolute difference of at least three CRS-R total-score points between the AI-standardized score and the adjudicated reference, or as a diagnostic shift across the UWS and MCS boundary. For such events, the study team will review input quality, EEG artifacts, rater disagreement, clinical confounders, model uncertainty, and prespecified feature-attribution summaries. The distribution of systematic errors across etiology, chronicity, airway status, sedation exposure, and severe motor impairment will be reported.

### 2.9 Sample size

The retrospective development sample size will be justified separately from the prospective validation sample size, consistent with current guidance for clinical prediction model and AI model reporting (34, 37, 38). The final development report will state the number of unique patients, the number of assessment episodes, the number of episodes per patient, diagnostic-category frequencies, CRS-R subscale-level distributions, and the number of candidate and retained predictors. The model-development strategy will not rely on a simple events-per-variable rule. Instead, the final feature space and model complexity will be constrained through prespecified feature grouping, training-fold-only feature selection, penalization or tree-complexity limits when applicable, and patient-level nested cross-validation. Learning-curve or resampling summaries will be used to assess whether performance estimates remain unstable as development data are increased.

The prospective validation sample size is based on the participant-level primary endpoint. Assuming a mean participant-level reduction of 1.0 CRS-R point in absolute total-score error after AI-assisted standardization, a standard deviation of participant-level paired differences of 2.5, a two-sided alpha of 0.05, and 80% power, at least 98 evaluable participants are required. Allowing approximately 15% attrition due to unusable EEG, incomplete paired assessments, missing videos, or withdrawal, the planned recruitment target is 120 participants.

### 2.10 Statistical analysis

All analyses will be prespecified and performed after prospective database lock. The primary analysis population will include participants with two blinded bedside CRS-R assessments, usable EEG according to protocol, and an adjudicated reference score. A feasibility population will include all consented participants.

Baseline characteristics will be summarized as mean and standard deviation or median and interquartile range for continuous variables, and as counts and percentages for categorical variables. The primary analysis will summarize *D*_*i*_ and test whether the mean participant-level error reduction differs from zero using a paired test appropriate to the empirical distribution. A linear mixed-effects model with participant as a random effect and method and rater as fixed effects will be used as a secondary sensitivity analysis to estimate rater-level effects. Intraclass correlation coefficients, mean absolute error, root mean square error, and Bland-Altman plots will be reported with 95% confidence intervals.

Diagnostic-category agreement will be assessed with weighted kappa, macro-averaged F1-score, sensitivity, specificity, and confusion matrices using the mapping rules in Table 1. Subscale-level performance will be assessed using ordinal agreement metrics, weighted kappa, and subscale-specific absolute error. Inter-rater variability before and after AI-assisted standardization will be compared using paired tests and mixed-effects models. The uncertainty module will be evaluated by testing whether high-uncertainty cases are enriched for large scoring error, rater disagreement, or reference-standard disagreement.

Confidence intervals for primary and major secondary metrics will be estimated using participant-level bootstrap resampling. Prespecified subgroup analyses will explore model performance across etiology, chronicity, tracheostomy status, severe motor impairment, and recent sedative exposure. Sensitivity analyses will exclude participants with substantial clinical changes between ratings, cases exceeding target timing windows, and cases with major sedation or airway changes during the assessment episode. A reduced-feature inference mode using report-level EEG variables only will also be tested.

### 2.11 Missing data

The adjudicated reference score will not be imputed. Missing clinical contextual covariates required for inference will be handled by the locked preprocessing pipeline, including explicit missingness indicators where applicable. Missing-data handling rules will be estimated or fixed within training folds during retrospective development and locked before prospective validation. If quantitative EEG features cannot be extracted but report-level EEG variables are available and the locked fallback pathway is triggered, the case will remain analyzable. Cases lacking both usable quantitative EEG and report-level EEG information will be counted as technical failures for the primary analysis and retained in feasibility summaries.

### 2.12 Data management and software environment

Study data will be entered into a secured electronic case report form with role-based access control and an audit trail. Personal identifiers will be stored separately from the analytic dataset. Each participant will be assigned a study identification code linking behavioral, clinical, EEG, video, and adjudication data.

The AI deployment package will be version-controlled, checksum-archived, and mounted on a secure institutional server. System downtime, failed inference, and fallback to manual review will be logged. The prospective validation pipeline will be executed in a locked software environment implemented in Python and R. Statistical analysis code will be finalized before outcome analysis and archived as a versioned study record.

## 3 Discussion

This study protocol addresses a practical limitation in the bedside assessment of DoC. Standardized behavioral examination remains central to diagnosis and clinical decision-making, but first-pass CRS-R scoring may be affected by arousal fluctuation, severe motor or language impairment, sensory limitations, sedative exposure, and inter-rater variability (1, 3, 11, 12). Repeated behavioral assessments reduce misclassification, yet they may not fully account for covert awareness or unstable responsiveness (6, 7, 13, 14). The proposed study therefore evaluates an AI-assisted standardization strategy as a human-supervised adjunct to CRS-R interpretation rather than as a replacement for clinical examination.

The main methodological contribution is the preservation of CRS-R subscale structure. Recent work highlights that CRS-R total scores, diagnostic thresholds, and subscale patterns do not carry identical clinical information across etiologies and assessment contexts (16–18). A direct total-score regression model could therefore produce an apparently accurate total score while obscuring the item-level pattern that supports diagnostic categorization. The planned two-stage architecture, with subscale-specific ordinal modules followed by rule-constrained integration, is intended to maintain clinically interpretable CRS-R profiles while still allowing multimodal standardization using EEG and contextual variables.

This protocol also responds to a translational gap in AI research for DoC. EEG and multimodal biomarkers increasingly show promise for detecting awareness, estimating prognosis, or characterizing brain-state dynamics, but many AI studies remain retrospective and focus on broad state classification rather than prospective validation of a locked, auditable decision-support workflow (5, 20–22, 30, 31). By locking the preprocessing pipeline, feature definitions, model parameters, uncertainty thresholds, and fallback logic before prospective enrollment, the study is designed to test whether the system can perform under real clinical constraints rather than under iterative model-development conditions.

Several design features are intended to reduce avoidable bias. Paired independent CRS-R assessments will allow direct comparison between raw inter-rater variation and post-standardization agreement. Blinding of bedside raters, EEG readers, adjudicators, and the statistical team should limit expectation effects and incorporation bias. The adjudicated reference will integrate structured behavioral observations, available video, nursing observations, medication exposure, airway status, body position, and other confounders, reflecting the clinical reality that a single bedside score may not capture the highest reproducible behavior. This design is aligned with SPIRIT-AI principles, which emphasize transparent reporting of intervention components, human-AI interaction, handling of errors, and procedures for monitoring algorithm performance (33).

The study has limitations. It is single-center, and its findings may be influenced by local patient mix, EEG equipment, assessment culture, rehabilitation workflow, and adjudication practice. The reference standard is expert-adjudicated but still behaviorally anchored, and it cannot fully resolve covert consciousness detected only by specialized task-based neuroimaging or electrophysiology (2, 4, 6). Video availability may vary according to consent and local policy. EEG artifacts and clinical instability are expected in this population, and fallback to report-level EEG variables may reduce model precision. Finally, because the system will operate in read-only mode, this validation study will assess standardization accuracy, feasibility, and safety signals, not whether AI-assisted scoring improves downstream treatment decisions or patient outcomes.

If successful, the study may provide a reproducible framework for human-supervised AI-assisted CRS-R score standardization. Its most plausible near-term role would be to flag cases requiring repeat assessment, expert review, or closer scrutiny of confounding conditions, particularly when model uncertainty is high or when standardized scores differ substantially from raw bedside scores. Such a use case would be consistent with the current maturity of EEG-based and AI-based approaches in DoC, where decision support and standardization are more defensible than autonomous diagnosis (1, 5, 30).

## 4 Ethics and dissemination

The study was approved by the Ethics Committee of Guangdong Second Traditional Chinese Medicine Hospital (approval number Z202603-004-01; review date March 25, 2026). The approval is valid from March 27, 2026 to March 26, 2027. The approved project title is “Application of an EEG-based multimodal artificial intelligence model in standardized CRS-R score assessment for patients with disorders of consciousness.” Written informed consent will be obtained from legally authorized representatives or immediate family members before prospective participation.

The study is registered in the Chinese Clinical Trial Registry (ChiCTR2600124984). The study will be conducted in accordance with applicable ethical requirements for human biomedical research. Protocol modifications, changes in principal investigator, early termination, serious adverse events, and other non-anticipated events affecting risk-benefit balance will be reported to the ethics committee according to local requirements.

Results will be submitted for peer-reviewed publication and presented at academic meetings. No identifiable participant information will be disclosed in publications or presentations.

## References

[1] Bodien YG Busl KM Chang CWJ Claassen J Gaspard N Gosseries O . Disorders of consciousness diagnosis, interventions, and prognostication for the intensivist: report of the 2025 ISICEM roundtable. Intensive Care Med. (2026) 52:42–62. doi: 10.1007/s00134-025-08224-1 41396553 PMC12852174

[2] Kazazian K Edlow BL Owen AM. Detecting awareness after acute brain injury. Lancet Neurol. (2024) 23:836–44. doi: 10.1016/S1474-4422(24)00209-6 39030043

[3] Threlkeld ZD Bodien YG Edlow BL. A scientific approach to diagnosis of disorders of consciousness. Handb Clin Neurol. (2025) 207:49–66. doi: 10.1016/B978-0-443-13408-1.00003-8 39986727

[4] Kazazian K Monti MM Owen AM. Functional neuroimaging in disorders of consciousness: towards clinical implementation. Brain. (2025) 148:2283–98. doi: 10.1093/brain/awaf075 39997570 PMC12233511

[5] Ridha M Kumar A Claassen J. Electrophysiology in disorders of consciousness. Handb Clin Neurol. (2025) 207:129–46. doi: 10.1016/B978-0-443-13408-1.00013-0 39986717

[6] Bodien YG Allanson J Cardone P Bonhomme A Carmona J Chatelle C . Cognitive motor dissociation in disorders of consciousness. N Engl J Med. (2024) 391:598–608. doi: 10.1056/NEJMoa2400645 39141852 PMC7617195

[7] Edlow BL Menon DK. Covert consciousness in the ICU. Crit Care Med. (2024) 52:1414–26. doi: 10.1097/CCM.0000000000006372 39145701

[8] Carroll EE Shen Q Kansara V Casson N Michalak A Niesvizky-Kogan I . Sleep spindles as a predictor of cognitive motor dissociation and recovery of consciousness after acute brain injury. Nat Med. (2025) 31:1578–85. doi: 10.1038/s41591-025-03578-x 40033114 PMC13519939

[9] Young MJ Edlow BL Bodien YG. Covert consciousness. NeuroRehabilitation. (2024) 54:23–42. doi: 10.3233/NRE-230123 38217619 PMC10898447

[10] Bodien YG Fecchio M Gilmore N Freeman HJ Sanders WR Meydan A . Multimodal biomarkers of consciousness in acute severe traumatic brain injury. J Neurotrauma. (2026) 43:373–86. doi: 10.1177/08977151251377469 41182259 PMC13191150

[11] Campagnini S Llorens R Navarro MD Colomer C Mannini A Estraneo A . Which information derived from the Coma Recovery Scale-Revised provides the most reliable prediction of clinical diagnosis and recovery of consciousness? A comparative study using machine learning techniques. Eur J Phys Rehabil Med. (2024) 60:190–7. doi: 10.23736/S1973-9087.23.08093-0 38193722 PMC11114154

[12] Aubinet C Gillet A Regnier A. Disorders of consciousness, language and communication following severe brain injury. Psychol Belg. (2025) 65:169–88. doi: 10.5334/pb.1381 40520820 PMC12164750

[13] Wannez S Heine L Thonnard M Gosseries O Laureys S Coma Science Group collaborators. The repetition of behavioral assessments in diagnosis of disorders of consciousness *Ann Neurol*. (2017) 81:883–9. doi: 10.1002/ana.24962 28543735

[14] Wang J Hu X Hu Z Sun Z Laureys S Di H. The misdiagnosis of prolonged disorders of consciousness by a clinical consensus compared with repeated coma-recovery scale-revised assessment. BMC Neurol. (2020) 20:343. doi: 10.1186/s12883-020-01924-9 32919461 PMC7488705

[15] Cortese MD Riganello F Arcuri F Pugliese ME Lucca LF Dolce G . Coma recovery scale-r: variability in the disorder of consciousness. BMC Neurol. (2015) 15:186. doi: 10.1186/s12883-015-0455-5 26450569 PMC4599033

[16] Weaver JA Cogan AM Kozlowski AJ Grady-Dominguez P O'Brien KA Bodien YG . Interpreting change in disorders of consciousness using the Coma Recovery Scale-Revised. J Neurotrauma. (2024) 41:e1996–2008. doi: 10.1089/neu.2023.0567 38613812 PMC11386986

[17] Grady-Dominguez P Bodien YG O'Brien KA Giacino JT Weaver JA. Evaluating the hierarchy of rating scale categories for the Coma Recovery Scale-Revised in non-traumatic brain injury: a Rasch analysis. Neurorehabil Neural Repair. (2026) 40:37–48. doi: 10.1177/15459683251399142 41451652

[18] Caselli S Leonardi M Magnani FG Cacciatore M Barbadoro F Ippoliti C . Comparing the different sets of item-level diagnostic criteria of the Coma Recovery Scale-Revised (CRS-R): a measurement-based approach driven by Rasch analysis. Arch Phys Med Rehabil. (2025) 106:1028–38.e8. doi: 10.1016/j.apmr.2024.12.009 39706237

[19] Eshraghi R Yazdani MS Bahrami A Amani-Beni R Darouei B Mokhtari M . Advanced neuromonitoring techniques for medical and neurological ICU patients. Brain Res Bull. (2025) 230:111513. doi: 10.1016/j.brainresbull.2025.111513 40829686

[20] Lee M Laureys S. Artificial intelligence and machine learning in disorders of consciousness. Curr Opin Neurol. (2024) 37:614–20. doi: 10.1097/WCO.0000000000001322 39498844

[21] Yang H Wu H Kong L Luo W Xie Q Pan J . Precise detection of awareness in disorders of consciousness using deep learning framework. Neuroimage. (2024) 290:120580. doi: 10.1016/j.neuroimage.2024.120580 38508294

[22] Dong W Yang Y Wu T Gao X Lin Y He J. Objective assessment of disorders of consciousness based on EEG temporal and spectral features. Int J Neural Syst. (2026) 36:2550067. doi: 10.1142/S0129065725500674 40985067

[23] Krumm L Kranz DD Halimeh M Nelde A Amorim E Zafar S . Toward a universal map of EEG: A semantic, low-dimensional manifold for EEG classification, clustering, and prognostication. Ann Neurol. (2025) 98:357–68. doi: 10.1002/ana.27260 40539771 PMC12278028

[24] Qu S Wu X Huang L Zhou Y Sun Q Zeng F. Diagnosis of disorders of consciousness using nonlinear feature derived EEG topographic maps via deep learning. Sci Rep. (2026) 16:7417. doi: 10.1038/s41598-026-36733-6 41644597 PMC12929573

[25] Della Bella GA Zang D Gui P Mateos DM Sitt JD Bekinschtein TA . Detection of EEG dynamic complex patterns in disorders of consciousness. Commun Biol. (2025) 8:1204. doi: 10.1038/s42003-025-08666-9 40796934 PMC12344011

[26] Zhang W Shi X Li M Zhang L Zhang R Wu X . Assess the level of consciousness in patients with disorders of consciousness by combining resting-state and auditory-evoked EEG. Front Neurosci. (2025) 19:1613356. doi: 10.3389/fnins.2025.1613356 41306427 PMC12645389

[27] Wang N Chai X Song J He Y He Q Zhang T . Motor intention quantization for patients with disorders of consciousness by multimodal BCI combining electroencephalography and functional near-infrared spectroscopy. CNS Neurosci Ther. (2025) 31:e70679. doi: 10.1002/cns.70679 41351188 PMC12680568

[28] Núñez P Tewarie P Rodríguez-González V Alnagger NLN van der Lande GJM Vitello MM . Altered electrophysiological meta-state dynamics in disorders of consciousness. Neuroimage. (2025) 323:121519. doi: 10.1016/j.neuroimage.2025.121519 41072637

[29] Manasova D Belloli LML Rosenfelder MJ Willacker L Fló Rama E Valota C . Multimodal multicentre investigation of diagnostic and prognostic markers in disorders of consciousness. Brain. (2026) 149:1381–95. doi: 10.1093/brain/awaf412 41499248 PMC13058464

[30] Bonanno M Cardile D Liuzzi P Celesti A Micali G Corallo F . Can artificial intelligence improve the diagnosis and prognosis of disorders of consciousness? A scoping review. Front Artif Intell. (2025) 8:1608778. doi: 10.3389/frai.2025.1608778 40520948 PMC12162576

[31] Narayanan A Magee WL Siegert RJ. Machine learning and network analysis for diagnosis and prediction in disorders of consciousness. BMC Med Inform Decis Mak. (2023) 23:41. doi: 10.1186/s12911-023-02128-0 36855149 PMC9972731

[32] Tinti L Lawson T Molteni E Kondziella D Rass V Sharshar T . Research considerations for prospective studies of patients with coma and disorders of consciousness. Brain Commun. (2024) 6:fcae022. doi: 10.1093/braincomms/fcae022 38344653 PMC10853976

[33] Rivera SC Liu X Chan AW Denniston AK Calvert MJ. Guidelines for clinical trial protocols for interventions involving artificial intelligence: the SPIRIT-AI extension. BMJ. (2020) 370:m3210. doi: 10.1136/bmj.m3210 32907797 PMC7490785

[34] Collins GS Moons KGM Dhiman P Riley RD Beam AL Calster BV . TRIPOD+AI statement: updated guidance for reporting clinical prediction models that use regression or machine learning methods. BMJ. (2024) 385:e078378. doi: 10.1136/bmj-2023-078378 38626948 PMC11019967

[35] Raveendran S Kenchaiah R Kumar S Sahoo J Farsana MK Chowdary Mundlamuri R . Variational mode decomposition-based EEG analysis for the classification of disorders of consciousness. Front Neurosci. (2024) 18:1340528. doi: 10.3389/fnins.2024.1340528 38379759 PMC10876804

[36] Raveendran S Kala S Ramakrishnan AG Kenchaiah R Sahoo J Kumar S . Functional connectivity in EEG: a multiclass classification approach for disorders of consciousness. Front Neurosci. (2025) 19:1550581. doi: 10.3389/fnins.2025.1550581 40224645 PMC11985804

[37] Riley RD Ensor J Snell KIE Harrell FE Martin GP Reitsma JB . Calculating the sample size required for developing a clinical prediction model. BMJ. (2020) 368:m441. doi: 10.1136/bmj.m441 32188600

[38] Riley RD Ensor J Snell KIE Archer L Whittle R Dhiman P . Importance of sample size on the quality and utility of AI-based prediction models for healthcare. Lancet Digit Health. (2025) 7:100857. doi: 10.1016/j.landig.2025.01.013 40461350

