# Supplementary material for: Prospective validation protocol for artificial intelligence-assisted standardization of Coma Recovery Scale-Revised scores using electroencephalography and clinical data in disorders of consciousness
Source: Front Med (Lausanne). 2026 Aug 3;13:1904678. doi: 10.3389/fmed.2026.1904678 (PMC13478918; doi:10.3389/fmed.2026.1904678)
Supplement: Supplementary file 1 [file Data_Sheet_1.pdf]

Supplementary Table S1. SPIRIT and SPIRIT-AI Checklist

Prospective validation protocol for artificial intelligence-assisted standardization of CRS-R scores using EEG and clinical data in disorders of consciousness

This checklist maps the revised protocol to core SPIRIT reporting domains and AI-specific SPIRIT-AI extension items. Page and line numbers should be updated after final typesetting.

**Checklist domain**	**Item addressed in revised protocol**	**Manuscript location**
Title	Identifies the protocol as prospective validation of an AI-assisted CRS-R score standardization system.	Title; Abstract
Trial registration	Provides Chinese Clinical Trial Registry name, unique identifier, URL, and ethics approval details.	Abstract; Clinical Trial Registration; Ethics and Dissemination
Protocol version / status	Study is described as a future prospective validation following retrospective model development.	Methods and Analysis
Funding	Funding sources and funder non-involvement are stated.	Funding
Roles and responsibilities	Author contributions, clinical oversight, data management, and statistical analysis responsibilities are described.	Author Contributions; Data management and software environment
Background and rationale	Explains DoC diagnostic uncertainty, CRS-R variability, EEG rationale, neuromonitoring context, and AI translational gap.	Introduction
Objectives	Defines primary and secondary aims.	Study design and setting
Study design	Two-stage retrospective model development followed by prospective blinded clinical validation.	Study design and setting; Figure 1
Study setting	Single-center implementation at Guangdong Second Traditional Chinese Medicine Hospital.	Study design and setting
Eligibility criteria	Inclusion and exclusion criteria are listed.	Study population
Intervention / AI system	Describes the locked multimodal AI standardization system, subscale ordinal modules, rule-constrained integration, uncertainty logic, and explanations.	Model architecture; Inference workflow
Outcomes	Defines participant-level primary endpoint and secondary endpoints, including exploratory day-14 consciousness improvement.	Outcome measures
Participant timeline	Specifies paired CRS-R assessments, EEG timing, video recording, offline AI inference, and adjudication.	Assessment workflow
Sample size	Separates retrospective model-development justification from prospective validation power calculation.	Sample size
Recruitment	Adult inpatients with acquired DoC will be screened during recruitment.	Study population
Allocation	Not applicable: this is a prospective validation study with read-only AI inference and no treatment allocation.	Prospective clinical validation
Blinding	Bedside raters, EEG readers, adjudicators, and statistical team blinding are described.	Rater training, standardization, and blinding

Continued on next page

Checklist domain	Item addressed in revised protocol	Manuscript location
Data collection	Behavioral, EEG, medication, airway, treatment timing, arousal, and contextual variables are specified.	Clinical contextual variables; EEG acquisition and preprocessing; Sedation and medication handling
Data management	Electronic case report forms, role-based access control, audit trail, and de-identification are described.	Data management and software environment
Statistical methods	Primary participant-level analysis, rater-level sensitivity analysis, agreement metrics, bootstrap CIs, subgroup and sensitivity analyses are specified.	Statistical analysis
Monitoring / error review	Serious prediction discrepancy events and error-review workflow are prespecified.	Error analysis
Harms / safety	AI is read-only during validation and will not determine treatment allocation.	Prospective clinical validation; Ethics and Dissemination
Ethics and consent	Ethics approval, approval validity, and representative consent are stated.	Ethics and Dissemination; Ethics Statement
Confidentiality	De-identification and separate storage of identifiers are described.	Data management and software environment
Dissemination	Peer-reviewed publication and academic presentation are planned.	Ethics and Dissemination
Data and code availability	Future de-identified data and code-availability conditions are stated.	Data Availability Statement; Code Availability Statement
SPIRIT-AI: AI in title/abstract	Title and abstract identify an AI-assisted clinical validation protocol.	Title; Abstract
SPIRIT-AI: intended use	The AI system is intended to standardize CRS-R scoring and flag cases for review, not to replace clinical judgment.	Introduction; Prospective clinical validation; Discussion
SPIRIT-AI: users and setting	Users and setting are specified as trained clinicians and offline read-only validation in neurological rehabilitation workflow.	Rater training; Prospective clinical validation
SPIRIT-AI: input data	Inputs include raw CRS-R subscale vector, EEG signal/features, and clinical context vector.	Clinical contextual variables; EEG acquisition; Algorithm 1
SPIRIT-AI: poor-quality or unavailable input data	EEG QC failure, fallback to report-level EEG variables, and technical-failure handling are specified.	EEG acquisition and preprocessing; Missing data; Algorithm 1
SPIRIT-AI: human-AI interaction for inputs	Offline AI inference uses rater-specific CRS-R scores, EEG data, and context; system is read-only during validation.	Assessment workflow; Prospective clinical validation
SPIRIT-AI: required expertise	Raters complete CRS-R training; EEG readers and adjudication experts are blinded and trained for their roles.	Rater training, standardization, and blinding
SPIRIT-AI: AI output	Outputs include standardized subscales, total score, diagnosis, uncertainty score, review flag, and explanation object.	Model architecture; Algorithm 1
SPIRIT-AI: contribution to decision-making	Outputs are evaluated against reference scores and do not determine treatment allocation during validation.	Prospective clinical validation; Outcome measures
SPIRIT-AI: versioning and locking	Model parameters, preprocessing rules, thresholds, software versions, and checksums are locked before prospective use.	Internal validation and model locking; Data management and software environment
SPIRIT-AI: model updating	No online learning, threshold tuning, or parameter updating is allowed during prospective validation.	Internal validation and model locking

Continued on next page

Checklist domain	Item addressed in revised protocol	Manuscript location
SPIRIT-AI: performance monitoring	Uncertainty flags, serious discrepancy events, and systematic error distributions are reviewed.	Error analysis; Statistical analysis
SPIRIT-AI: explainability	Prespecified additive feature attribution supports transparency and error review.	Inference workflow
SPIRIT-AI: error case analysis	Serious prediction discrepancy events are defined and reviewed for artifacts, rater disagreement, confounders, uncertainty, and attribution summaries.	Error analysis
SPIRIT-AI: data privacy / governance	De-identification, access control, audit trail, and institutional governance for data/code sharing are described.	Data management; Data Availability Statement; Code Availability Statement
